# The Malay version of the caregiver assessment of function and upset instrument (Malay-CAFU): a translation and validation study among informal stroke caregivers

**DOI:** 10.1186/s12889-023-15076-1

**Published:** 2023-01-30

**Authors:** Nurfaten Hamzah, Kamarul Imran Musa, Muhammad Hibatullah Romli, Xin Wee Chen, Mohd Zulkifli Abdul Rahim, Jafri Malin Abdullah, Mohd Azmi Suliman, Mohd Ismail Ibrahim, Tengku Alina Tengku Ismail, Iliatha Papachristou Nadal, Suresh Kumar Kamalakannan

**Affiliations:** 1 Department of Neurosciences, School of Medical Sciences, Health Campus, Universiti Sains Malaysia, Kubang Kerian, Kelantan Malaysia; 2 Brain and Behaviour Cluster, School of Medical Sciences, Health Campus, Universiti Sains Malaysia, Kubang Kerian, Kelantan Malaysia; 3 Department of Community Medicine, School of Medical Sciences, Health Campus, Universiti Sains Malaysia, Kubang Kerian, Kelantan Malaysia; 4 Department of Nursing and Rehabilitation, Faculty of Medicine and Health Sciences, Universiti Putra Malaysia, Serdang, Selangor Malaysia; 5 Malaysian Research Institute of Ageing (MyAgeingTM), Universiti Putra Malaysia, Serdang, Selangor Malaysia; 6 Department of Public Health Medicine, Faculty of Medicine, Universiti Teknologi MARA, Sungai Buloh Campus, Selangor Malaysia; 7 Interdisciplinary Health Sciences Unit, School of Health Sciences, Health Campus, Universiti Sains Malaysia, Kubang Kerian, Kelantan Malaysia; 8 Department of Neurosciences & Brain Behaviour Cluster, Hospital Universiti Sains Malaysia, Health Campus, Universiti Sains Malaysia, Kubang Kerian, Kelantan Malaysia; 9 Department of Non-Communicable Diseases and Epidemiology, Faculty of Epidemiology and Population Health, London School of Hygiene and Tropical Medicine, Keppel Street, London, UK; 10 Department of Clinical Research, Faculty of Infectious and Tropical Diseases, London School of Hygiene and Tropical Medicine, Keppel Street, London, UK

**Keywords:** Stroke survivors, Informal caregivers, Translation, Validation, Reliability, Explanatory factor analysis, Caregiver assessment of function and upset

## Abstract

**Background:**

Post-stroke complications affect the informal caregivers equally as the stroke survivors, especially those who have a moderate to worst prognosis in functional capacity recovery. Caregiver Assessment of Function and Upset (CAFU) is one of the common tools used in both research and clinical practice to measure the patient’s dependency level and the stroke caregivers' upset level.

**Objective:**

This study aimed to translate and validate the CAFU instrument into the Malay language and test the validity and reliability of the CAFU among informal stroke caregivers in Malaysia.

**Methods:**

A standard forward-backward translation method was employed to translate CAFU. Subsequently, 10 expert panels were included in the validation process, and thereafter reliability testing was conducted among 51 stroke caregivers. The validation of the instrument was determined by computing the content validity indices (CVIs), and we used the Cronbach’s alpha method to explore the internal consistency of the overall score and subscales scores of the Malay-CAFU. Finally, the explanatory factor analysis used principal component extraction and a varimax rotation to examine construct validity.

**Results:**

All items of the Malay-CAFU had satisfactory item-level CVI (I-CVI), with values greater than 0.80, and the scale-level CVI (S-CVI) was 0.95. These results indicate that the Malay-CAFU had good relevancy. The internal consistency for the reliability test showed a Cronbach’s alpha value of 0.95 for the overall score. The eigenvalues and scree plot supported a two-factor structural model of the instrument. From the explanatory factor analysis, the factor loadings ranged from 0.82 to 0.90 and 0.56 to 0.83, respectively.

**Conclusion:**

The Malay-CAFU questionnaire is a valid and reliable instrument to assess the dependence level of stroke survivors and the upset level of informal stroke caregivers in Malaysia.

**Supplementary Information:**

The online version contains supplementary material available at 10.1186/s12889-023-15076-1.

## Introduction

Stroke causes significant morbidity and mortality in both developed and developing countries, and it is one of the leading causes of long-term disability and dependency in daily activities worldwide [1, 2]. In Malaysia, stroke is the third leading cause of mortality, and almost half of the stroke survivors have experienced functional dependence at the time of hospital discharge following their first episode of stroke [3–5]. Stroke survivors often suffer from different degrees of physical, functional, or cognitive disabilities requiring acute inpatient treatment and extended care at home, particularly those with moderate to severe disabilities [2, 6].

Caregivers can be formal or informal. Healthcare professionals who are paid for the care and support they give to patients or clients are considered formal caregivers. These professions include nurses, personal support workers, rehabilitation specialists, and doctors [7]. An informal caregiver is an individual who provides unpaid care and is responsible for the patient’s post-care at home [8, 9]. As cultural values and norms influence people to care for their closest family members, the family usually provides the first line of support for informal care [10] and can be seen as a practical way to reduce the costs of healthcare services while at the same time supporting the common preferences among patients to receive care at home and in their familiar environment [11].

Most stroke caregivers in Malaysia are informal caregivers, comprising family members, typically the spouse. Informal caregivers play an important role in providing assistance, rehabilitation, nursing care, and helping stroke survivors at home [12, 13]. These include assisting stroke survivors with basic activities of daily living (ADL), such as feeding, mobilizing, bathing, dressing, toileting, and grooming; and instrumental activities of daily living (IADL), such as managing finances, transportation, shopping, meal preparation, housekeeping, managing communication, and handling medication [12]. Thus, it has been common for caregivers to be asked by treating healthcare professionals like doctors and rehabilitation physicians to evaluate the functional performance of stroke survivors [14]. Therefore, the caregiver’s accurate and precise evaluation is a critical component that can drive the intervention plan for stroke survivors.

 However, the caregiving process for stroke survivors may result in a psychological burden for the caregiver. Particularly, when the caregiver’s own needs, such as their physical, emotional, social, and financial needs, were poorly addressed during the survivor’s recovery [6, 15, 16]. Caregivers may experience emotional upset as well as mental health issues such as stress, anxiety, and depression [6, 17]. These psychological burdens affect the caregiver’s quality of life [18] and lead to the abandonment of the caregiving role [8].

The assessment of stroke survivors’ dependence and caregivers’ upset levels is lacking in Malaysia, partly due to the unavailability of reliable and validated tools in the Malay language (Malaysia’s national language). An example of such a tool is the Caregiver Assessment of Function and Upset (CAFU) instrument that assessed the caregiver’s appraisal of physical dependence level in 15 daily activities of a patient and the caregiver’s upset level when assisting in each area of the daily activities [19]. Another instrument, the Zarit Burden Interview (ZBI), is commonly used to evaluate caregiver burden in clinical and research settings [8]. ZBI, on the other hand, only assesses the caregiver’s caregiving burden, whereas CAFU assesses both the patient’s functional dependence and the caregiver’s burden in the caregiving role. Since the aim of our study was to investigate both the stroke survivor’s dependency level and the caregiver’s upset, CAFU is more suitable for our study setting. Our study aimed to investigate the stroke survivor’s dependency level and the caregiver’s upset; therefore, CAFU suits this study. Specifically, this study involves a) the translation of the original CAFU instrument into the Malay language (Malay-CAFU) and b) the investigation of the validity and reliability of the Malay-CAFU among informal stroke caregivers in Malaysia. One of the primary benefits of this validated Malay-CAFU is that it can be applied in a future study to understand better the dependency level of stroke survivors and the upset level of their caregivers. Consequently, this information can be applied in developing stroke rehabilitation interventions to reduce mortality and morbidity among stroke survivors and improve caregiving competence and stress coping in informal caregivers.

## Methods

**Fig. 1:**
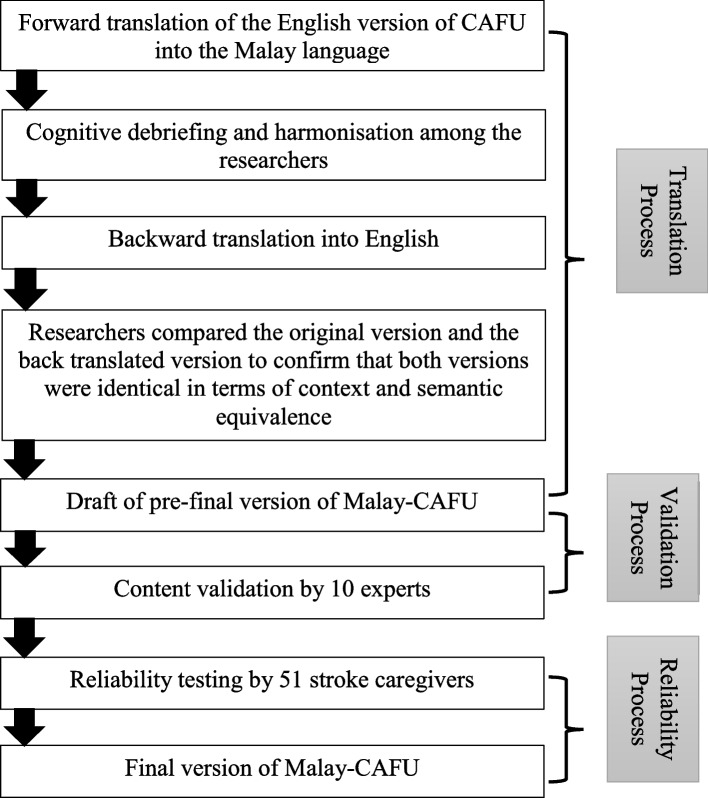
Workflow for the translation, validation, and reliability process of the Caregiver Assessment of Function and Upset instrument into the Malay language

The study was cross-sectional, with a source population of 10 experts and 51 stroke caregivers. This study began in September 2020 and ended in August 2021. There were three stages involved in this study workflow: (1) translation, (2) validation, and (3) reliability (refer Fig. 1).

### Overview of the CAFU instrument

Gitlin et al. developed the Caregiver Assessment of Functional and Upset instrument, a 15-item instrument, on a sample of dementia patients and their caregivers to evaluate the caregiver’s assessment of the physical dependence level in 15 daily activities of patients as well as the caregiver’s upset level when assisting in each area of the daily activities [19]. A measure that reflects the physical dependence of people with dementia and caregiver reactions to providing assistance would be useful in guiding an intervention approach that addresses both the patient’s physical functional needs and the caregiver’s emotional upset. A measure that examines the functioning of both the caregiver and the patient is particularly crucial in the context of recent research showing that the quality of life for dementia patients is impacted by caregiver assessments and dementia management strategies [19].

This instrument consists of two subscales: (a) IADLs, eight items (telephone, shopping, meal preparation, housework, laundry, travelling, medication, and finances); and (b) ADLs, seven items (getting in and out of bed, eating, bathing, dressing upper body, dressing lower body, toileting, and grooming). Each item consists of the physical dependence level of the patient and the upset level of the caregiver. For the physical dependence level, the respondents rated each of the items using a 5-point Likert scale ranging from 1 (complete help) to 5 (no physical help). Whereas, for the upset level of the caregiver, the respondents gave a rate using a 4-point Likert scale ranging from 0 (not at all) to 4 (extremely). Previous studies showed that the CAFU instruments have good internal consistency and acceptable convergent and discriminant validity for both patient dependency and caregiver upset level measures. Written permission to translate the CAFU instrument was obtained from the developer.

### Translation of the Malay-CAFU

The translation process aimed to develop an equivalent version of the original instrument for use among Malaysian respondents. We followed the cross-cultural adaptation guideline to ensure that the translated version was identical to the original version [20]. The guideline provides details for the forward translation and backward translation processes. During the forward translation process, three translators (two certified professional translators and one native speaker) translated the English version of CAFU to the Malay version of CAFU (Malay-CAFU). Then, the main researchers and the translators analysed the resulting translations via a Zoom teleconference application (cognitive debriefing) and the harmonisation of the instrument to ensure that the translated items resembled those of the original version. The final consensus produced a draft version of the Malay-CAFU. This harmonised version was then backward translated into English by another certified professional translator. Finally, the researchers compared the backward translated version with the original English version to ensure that both versions were identical in terms of context and semantic equivalence, and to derive the pre-final Malay-CAFU instrument [21, 22]. All certified professional translators involved in this process (except for native speakers) were permanent staff at the Language, Literacy and Translation Centre, Health Campus, Universiti Sains Malaysia, Kelantan. 

### Validation of the Malay-CAFU

The Malay-CAFU underwent a content validity assessment by a panel of 10 experts. The aim of the assessment is a) to confirm that the translated instrument measures what it is supposed to measure, b) to assess its appropriateness and c) to assess its relevance with the study objectives [23]. The content validity was assessed using Google Forms created by the researchers, which were then distributed to the 10 panel experts, composed of five healthcare professionals who have had extensive clinical experience in post-stroke management and five caregivers (refer Supplementary material 1). The informal caregivers were considered experts as they had experience in caregiving. They were given both the original and the translated versions of the instrument and were asked to rate the relevancy of the translation on a four-point scale: 1 = item is not relevant; 2 = item is somewhat relevant; 3 = item is quite relevant; and 4 = item is highly relevant [23]. Subsequently, the content validity indices (CVIs) for item-level (I-CVI) and scale-level (S-CVI) were computed.

### Reliability test of the Malay-CAFU

The Malay-CAFU then underwent pilot testing for reliability using an internal consistency measure (Cronbach’s alpha coefficients). The study participants were from Malaysia's Malay-predominant states – Kelantan and Terengganu. For reliability testing, this study requires a sample size of 39 caregivers to achieve the expected Cronbach’s alpha of 0.80, the precision of 0.10 and at a 95% of confidence interval [24]. With an expected dropout rate of 20% taken into account, the final sample size calculated was 49 informal caregivers [24]. The source population was 51 stroke caregivers who were recruited from Hospital Raja Perempuan Zainab II (HRPZ), Kelantan, Hospital Universiti Sains Malaysia (HUSM), Kelantan and Hospital Sultanah Nur Zahirah (HSNZ), Terengganu. The following criteria were used to select the respondents: (i) age 18 years or older; (ii) legal guardian of the stroke survivor; (iii) taking care of the stroke survivor at the time of the study; (iv) ability to communicate in Malay; and (v) Malaysian citizenship. Respondents were excluded if they (i) refused to participate; (ii) did not live with the stroke survivor; and (iii) had cognitive problems.

### Statistical analysis

Data from the Google Form data were converted to Microsoft Excel, and all statistical analysis were conducted in IBM SPSS Statistics 26 software. The descriptive analysis for the demographic characteristics was summarized using the mean and standard deviation for numerical data, and categorical data was expressed using frequency (n) and percentages (%).

For the Content Validation Index (CVI), the relevance scores from the experts were recoded as 1 (relevance scale of 3 or 4) and 0 (relevance scale of 1 or 2) [25]. The CVI was computed to produce two measures: (1) the content validity of individual items (I-CVI); and (2) the content validity of the overall scale using the averaging calculation method (S-CVI). S-CVI was calculated using the following two formulas [25]:

I-CVI = (agreed item) / (number of participants).

S-CVI = (sum of I-CVI scores) / (number of item).

The S-CVI should be 0.80 to be considered acceptable for content validity, and the translated instruments were ready to be used for research purposes [26]. Items with an I-CVI greater than 0.80 can be retained, whereas I-CVIs between 0.70 and 0.79 should be revised and I-CVIs less than 0.70 should be rejected [27].

The internal consistency was measured by calculating the Cronbach’s alpha for the overall score and subscale scores. Cronbach’s alpha values greater than 0.70 were considered acceptable and showed high internal consistency [28]. The construct validity was investigated by performing an explanatory factor analysis on the dependence ratings with principal component extraction and a varimax rotation. The Kaiser-Meyer-Olkin (KMO) coefficient and the Bartlett’s test of sphericity were used to evaluate the data’s appropriateness for explanatory factor analysis. The KMO should be greater than 0.60, and the Bartlett’s test of sphericity is acceptable at *p* < 0.05 [29]. The number of factors to extract was determined using the eigenvalues (must be greater than 1) and the Scree plot. Items that have factor loadings greater than 0.40 would be kept [22].

## Results

The CVIs of the Malay-CAFU were computed for all individual items (I-CVI) and the overall scale (S-CVI) are reported (refer Supplementary material 2). All items in the IADL and ADL subscales showed excellent I-CVI results (values ranging from 0.80 to 1.00); hence all items were retained in the instrument. While, the value for S-CVI equals 0.95.

Table 1 describes the socio-demographic characteristics of 51 stroke caregivers participating in the reliability test. The age of the stroke caregivers ranged from 18 to 69 years, with a mean of 36.96 (14.36). The majority of the respondents were recruited from HUSM (45.10%), female gender (60.78%), married (70.59%), and studied up to the secondary level (70.59%). Most of the caregivers were stroke survivors’ children (72.55%).

**Table 1:** Socio-demographics characteristics of the stroke caregivers in the reliability testing process (*n* = 51)

	Frequency (%)	Mean (SD)
**Age**		36.96 ± 14.36
**Study sites**		
HRPZ	20 (39.22)	
HUSM	23 (45.10)	
HSNZ	8 (15.68)	
**Gender**		
Female	31 (60.78)	
Male	20 (39.22)	
**Education level**		
Primary	1 (1.96)	
Secondary	36 (70.59)	
Tertiary (College/ University)	14 (27.45)	
**Marital status**		
Single	15 (29.41)	
Married	36 (70.59)	
**Relationship with the stroke survivors**		
Child	37 (72.55)	
Spouse	12 (23.53)	
Sibling	2 (3.92)	

***SD*,** standard deviation**;** ***HRPZ***, Hospital Raja Perempuan Zainab II; ***HUSM***, Hospital Universiti Sains Malaysia; ***HSNZ***, Hospital Sultanah Nur Zahirah.

**Fig. 2:**
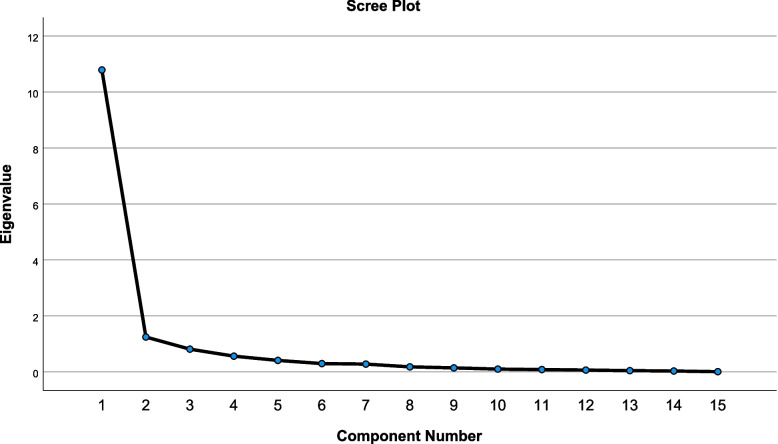
Scree plot

The KMO coefficient for this dataset was 0.85, and Bartlett’s test of sphericity was significant (*p* < 0.001). These findings demonstrated that the data were appropriate and sufficient for exploratory factor analysis. The eigenvalues (refer Table 2) and Scree plot (refer Fig. 2) were used to determine the number of factors to retain for factor analysis. Two factors appeared with eigenvalues greater than 1.00. The scree plot’s elbow also demonstrated the emergence of two factors. The loadings of the first factor and second factor ranged from 0.82 to 0.90 and 0.56 to 0.83, respectively (refer Table 3). The two-factor model had eigenvalues of 10.79 and 1.24, explaining 82.20% of the total variance (refer Table 2).

**Table 2:** Total variance explained (initial eigenvalues)

Component	Initial Eigenvalues			Comment
	Total	% of variance	Cumulative %	
1	10.79	71.92	71.92	Retain
2	1.24	8.28	82.20	Retain
3	0.81	5.40	85.60	Drop

**Table 3:** Principal Component Analysis of IADL and ADL dependence scores with varimax rotation

Variable	Two-factor model	
	Factor 1	Factor 2
**IADLs**		
Travelling		0.83
Finances		0.82
Laundry		0.79
Shopping		0.69
Housework		0.64
Meal preparation		0.61
Telephone		0.60
Medication		0.56
**ADLs**		
Grooming	0.90	
Toileting	0.88	
Getting in or out of bed	0.88	
Dressing below the waist	0.88	
Dressing above the waist	0.87	
Eating	0.84	
Bathing	0.82	

***IADL***, instrumental activities daily living; ***ADL***, activities of daily living.

The seven items loaded on the first factor reflected the caregiver’s assessment of the dependency level of stroke survivors in managing basic activities of daily living. The remaining eight items on the second factor reflected the caregiver’s assessment of stroke survivors’ dependency level in managing basic instrumental activities of daily living. The internal consistency for the overall instrument was 0.95. All of Cronbach’s alpha values for the overall instrument, subscale dependence of IADL, subscale upset of IADL, subscale dependence of ADL, and subscale upset of ADL were greater than 0.90 (refer Table 4). The Malay-CAFU instrument demonstrates high internal consistency for dependence and upset IADL and ADL subscales.

**Table 4:** Mean, SD and internal consistency values for the subscales of the Malay-CAFU

Subscale	Mean	Standard deviation	Cronbach’s alpha
**IADL**			
Dependence	5.66	1.91	0.91
Upset	0.70	1.11	0.95
**ADL**			
Dependence	5.35	2.08	0.97
Upset	0.79	1.13	0.97
		**Overall Cronbach’s alpha**	0.95

***IADL*** instrumental activities daily living, ***ADL*** activities of daily living.

The caregivers reported that stroke survivors needed more help to complete the IADLs compared to the ADLs, as the mean of the dependence IADL subscale was slightly higher than the dependence ADL subscale. In addition, the caregivers reported low average levels of upset, as the values were 0.70 and 0.79, respectively (refer Table 4).

## Discussions

A questionnaire is one of the well-known instruments for data collection; however, developing a new one might require extra cost and time [30]. Thus, an adaptation of the published instruments is recommended through translation and validation to ensure that the translated version is equivalent to the original version. In this study, we translated the 15-item CAFU from English into the Malay language (Malay-CAFU) and assessed its validity. The Malay-CAFU evaluates the informal caregiver assessment of the level of IADL and ADL assistance that stroke survivors need and the caregiver’s reaction to giving this assistance. The Malay-CAFU instrument has undergone rigorous and stringent translation, validation, and reliability processes according to well-established guidelines [20].

The process of translation and validation of the instrument was performed by certified professional translators and expert panels. In order to ensure that the content, concept, criterion, and semantic criteria matched and were equivalent to the original English version, the research team members’ review was essential for identifying mistakes and correcting them [31]. Regardless of sociodemographic differences, it should be easy for the general population in Malaysia to understand.

The validity of an instrument was determined by examining whether it measures what it was designed to measure [32]. CVI is commonly used by researchers to measure the validity of an instrument because it is easy to measure and understand, provides details for each item, and can be used to retain, revise, or delete the questionnaire items [28]. In our study, the CVIs were computed and reported at both the item and scale levels. The consistently excellent CVI range information for both the I-CVI and S-CVI above 0.80 demonstrated that the Malay-CAFU is relevant to the subscale, clear, and comprehensible for use by target users [30]. The internal consistency of the instrument measures the degree to which the items on the instrument are interrelated [33]. The Malay-CAFU exhibited excellent internal consistency, with almost similar values to the original (English-version) questionnaire [19]. Furthermore, explanatory factor analysis is used to produce stronger evidence that a measure’s factor structure is valid [31]. The explanatory factor analysis revealed that Malay-CAFU had two factors that measured the caregiver’s appraisal of the dependency level of stroke survivors on IADL and ADL. These findings were similar to the original English-version CAFU, where it demonstrated good internal consistency for both measures of dependency level and caregiver upset of IADL and ADL (values ranging from 0.80 to 0.91) and excellent construct validity for both dependency levels of IADL and ADL [19]. The analysis indicated that the content of the Malay-CAFU instrument is well adapted to the local context in Malaysia [21]. Therefore, the Malay-CAFU is useful to measure caregiver appraisals of the dependence level of stroke survivors and caregivers’ reactions when providing assistance in the Malay community of stroke survivors.

Several instruments are available for measuring caregivers’ reactions to assisting stroke survivors, but they do not evaluate caregiver responses to assisting in the IADL and ADL areas [19]. On the other hand, the Malay-CAFU provides a valid and reliable method for assessing 15 physical function areas of IADLs and ADLs as well as which functional dependence areas make caregivers upset. This method offers two benefits. First, it provides researchers and clinicians with a simple and quick technique to evaluate specific IADLs and ADLs that require caregiver assistance and also present a distressing problem. Second, the Malay-CAFU facilitates the development of targeted interventions to improve informal caregivers’ education and skill-training in the specific areas where assistance is offered, as well as methods for handling the upset that comes along with it. Thus, the Malay-CAFU may surpass other current functional status questionnaires that focus on self-reports or direct observation but do not take into consideration the caregiver’s role in providing help and the emotional effects that go along with it.

The Malay-CAFU measures an essential component of family life, the real situation of caregiving, and the caregiver's emotional state. Therefore, we believe that understanding caregivers assessment of the stroke survivors’ level of dependency and emotional reaction will give us insight into their daily care at home. Furthermore, in clinical settings, evaluating item-by-item reaction scores is useful when a) determining the emotional capacity of the caregiver when assisting the stroke survivor, and b) designing interventions that focus on specific areas of physical function causing the most significant challenges for the family [19].

This study concurred with other previous translation content validity studies in Malaysia where the translation was successful and the content was acceptable [34]. This study was conducted amidst the COVID-19 pandemic. It proved the feasibility of using teleconference for discussion and harmonisation (in the translation phase) and online data collection (through Google Form) for the translation and validation process. Therefore, the COVID-19 pandemic should not hinder researchers from developing and using valid instruments.

### Limitations

The present study assessed the validity of the Malay-CAFU in two Malay-predominant states and the demographic characteristics did not reflect the actual demographics of Malaysia due to the convenience sampling method used during the movement restrictions in the COVID-19 pandemic. However, the Malay language is the national language in Malaysia; hence, the tool is applicable to the local context. Future studies may assess the validity of the Malay-CAFU among the non-Malay adult Malaysian population, respondents from different socioeconomic statuses and Malay-speaking respondents from other countries such as Thailand, Singapore, Brunei and Indonesia that speak the Malay language.

## Conclusions

The Malay-CAFU (the Malay language CAFU) instrument has exhibited good validity, reliability, excellent CVI, both at the item and scale levels, and internal consistency. The Malay-CAFU would i) promote research that assesses the dependence level of stroke survivors and the upset level of stroke caregivers, and ii) guide an intervention approach that addresses both the physical functional needs of the stroke survivor and the emotional upset of the caregiver in Malaysia. 

## Supplementary Information

**Additional file 1.**

**Additional file 2.**

## Data Availability

The datasets used and/or analyzed during the study are available from the corresponding author upon reasonable request.

## Abbreviations

CAFU: Caregiver Assessment of Function and Upset
ADL: Activities of daily living
IADL: Instrumental activities of daily living
ZBI: Zarit Burden Interview
CVI: Content validity index
I-CVI: Item-level content validity index
S-CVI: Scale-level content validity index
KMO: Kaiser-Meyer-Olkin
HRPZ: Hospital Raja Perempuan Zainab II
HUSM: Hospital Universiti Sains Malaysia
HSNZ: Hospital Sultanah Nur Zahirah
