# Supplementary material for: The Malay version of the caregiver assessment of function and upset instrument (Malay-CAFU): a translation and validation study among informal stroke caregivers
Source: BMC Public Health. 2023 Jan 30;23:198. doi: 10.1186/s12889-023-15076-1 (PMC9885385; doi:10.1186/s12889-023-15076-1)
Supplement: Supplementary file 1 — Additional file 1. [file 12889_2023_15076_MOESM1_ESM.pdf]

Supplementary material 1

Demographic characteristics of the panel of experts of the content validation study (n=10)

Characteristics	Frequency (%)	Mean (SD)
**Age**		26 (2.05)
**Category**		
Healthcare Professional	5 (50)	
Caregiver	5 (50)	
**Gender**		
Female	9 (90)	
Male	1 (10)	
**Education level**		
School (Secondary)	1 (10)	
Tertiary (College/University)	9 (90)	

SD, standard deviation
