# Supplementary material for: The Malay version of the caregiver assessment of function and upset instrument (Malay-CAFU): a translation and validation study among informal stroke caregivers
Source: BMC Public Health. 2023 Jan 30;23:198. doi: 10.1186/s12889-023-15076-1 (PMC9885385; doi:10.1186/s12889-023-15076-1)
Supplement: Supplementary file 2 — Additional file 2. [file 12889_2023_15076_MOESM2_ESM.pdf]

Supplementary material 2

Ratings on the ADL and IADL subscales by 10 experts

	Rater 1	Rater 2	Rater 3	Rater 4	Rater 5	Rater 6	Rater 7	Rater 8	Rater 9	Rater 10	Experts in agreement	Items CVI
**IADL**												
Item 1	1	1	1	1	1	1	1	1	1	1	10	1
Item 2	1	1	1	1	1	1	1	1	1	0	9	0.90
Item 3	1	1	1	1	1	1	1	1	0	1	9	0.90
Item 4	1	1	1	1	1	1	1	1	1	1	10	1
Item 5	1	1	1	1	1	1	1	1	1	1	10	1
Item 6	1	1	1	1	1	1	1	1	1	0	9	0.90
Item 7	1	1	1	1	1	1	1	1	1	1	10	1
Item 8	1	1	1	1	1	1	1	1	1	0	9	0.90
**ADL**												
Item 9	1	1	1	1	1	1	1	1	1	0	9	0.90
Item 10	1	1	1	1	1	1	1	1	1	1	10	1
Item 11	1	1	1	1	1	1	1	1	1	1	10	1
Item 12	1	1	1	1	1	1	1	1	0	0	8	0.80
Item 13	1	1	1	1	1	1	1	1	1	0	9	0.90
Item 14	1	1	1	1	1	1	1	1	1	1	10	1
Item 15	1	1	1	1	1	1	1	1	1	1	10	1
											**S-CVI**	**0.95**

IADL, instrumental activities of daily living; **ADL**, activities of daily living; **CVI**, content validity index; **S-CVI**, scale-level content validity index
